# Simultaneous Measurement of Smoothened Entry Into and Exit From the Primary Cilium

**DOI:** 10.1371/journal.pone.0104070

**Published:** 2014-08-13

**Authors:** Jynho Kim, Elaine Y. C. Hsia, James Kim, Navdar Sever, Philip A. Beachy, Xiaoyan Zheng

**Affiliations:** 1 Departments of Biochemistry and Developmental Biology, Institute for Stem Cell Biology and Regenerative Medicine, Stanford University School of Medicine, Stanford, CA, United States of America; 2 Howard Hughes Medical Institute, Stanford University School of Medicine, Stanford, CA, United States of America; 3 Division of Hematology-Oncology, Hamon Center for Therapeutic Oncology Research, University of Texas Southwestern, Dallas, TX, United States of America; 4 Department of Anatomy and Regenerative Biology, George Washington University School of Medicine and Health Sciences, Washington, DC, United States of America; Indiana University School of Medicine, United States of America

## Abstract

Ciliary accumulation of signaling proteins must result from a rate of ciliary entry that exceeds ciliary exit, but approaches for distinguishing ciliary entry vs. exit are lacking. Using a photoconvertible fluorescent protein tag, we establish an assay that allows a separate but simultaneous examination of ciliary entry and exit of the Hedgehog signaling protein Smoothened in individual cells. We show that KAAD-cyclopamine selectively blocks entry, whereas ciliobrevin interferes initially with exit and eventually with both entry and exit of ciliary Smoothened. Our study provides an approach to understanding regulation of ciliary entry vs. exit of Hedgehog signaling components as well as other ciliary proteins.

## Introduction

The primary cilium, a microtubule-based organelle about 5 µm in length and 200 nm in diameter, projects from the surface of vertebrate cells to sense and interpret a variety of extracellular signals [1]. Previous work has suggested that the dynamic movement of receptors and other proteins into and out of cilia regulates the activity of signaling complexes that ultimately trigger responses in the cell [2]. It has been proposed that ciliary accumulation of signaling proteins results from a rate of ciliary entry that exceeds the rate of ciliary exit [3]. An understanding of signaling receptor trafficking into cilia is now emerging [4]; however, the mechanisms that underlie membrane protein removal from cilia and the regulation of this trafficking step remain largely unexplored. The major challenges arise not just from the small size of the primary cilium, but also from the difficulty of separately evaluating ciliary entry and exit of proteins that traffic through the primary cilium. In this context, development of new assays that can distinguish ciliary entry and exit are crucial to progress in understanding regulation of ciliary trafficking.

The Hedgehog (Hh) signaling pathway organizes pattern formation in a variety of embryonic tissues and functions post-embryonically in homeostatic processes. Hh pathway dysfunction thus can lead to birth defects such as holoprosencephaly (HPE) [5] or proliferative disorders such as the growth of malignant tumors [6]. The quiescent state of the Hh signaling pathway is maintained by Patched (Ptc) inhibition of Smoothened (Smo) [7]. This inhibition is lifted by binding of the extracellular Hh protein signal to Ptc, thus unleashing Smo activity and initiating a series of intracellular events that lead to changes in gene transcription. Recent studies have highlighted the importance of the primary cilium in transduction of mammalian Hh signals. Smo and other Hh pathway components in mammalian cells traffic through the primary cilium and accumulate upon Hh stimulation and Ptc inactivation [8]–[13]. Small molecules that either activate or inactivate Smo can also modulate signaling activity and ciliary localization of Smo [3], [14]. Given that accumulation of Smo in the primary cilium is one of the earliest hallmarks of Hh pathway activation, understanding regulation of Hh signal transduction depends critically on unveiling the molecular mechanism of Smo accumulation in the primary cilium.

Whereas ciliary entry has been emphasized as a critical point of regulation, the findings that Smo continuously shuttles into and out of the cilium in unstimulated cells [11], [15] and that levels of ciliary Smo eventually decrease once stimulation is terminated leave open the possibility that either entry or exit rates could be the target for regulation upon pathway engagement. Establishment of assays to separately monitor Smo ciliary entry and exit therefore could illuminate the general mechanism underlying protein accumulation in the primary cilium as well as shed light into how the Hh signaling pathway is regulated.

Here, we fuse a photoconvertible fluorescent protein, mEos2 [16], to the C-terminus of Smo (Smo-mEos2) and establish a live-cell imaging assay that allows a simultaneous examination of ciliary entry and exit of Smo in individual cells. Using this assay, we find that activated Smo enters and exits the cilium continuously with a ciliary retention half-life of approximately two hours. We also find that the small molecule KAAD-cyclopamine selectively blocks ciliary entry of Smo, whereas long-term exposure to ciliobrevin eventually interferes with both the ciliary entry and exit of Smo. Our study provides an approach to understanding the separate regulation of ciliary entry vs. exit of signaling proteins within and beyond the Hh pathway.

## Results and Discussion

### Ciliary entry and exit of Smo-mEos2 in SAG-treated cells

**Figure 1:**
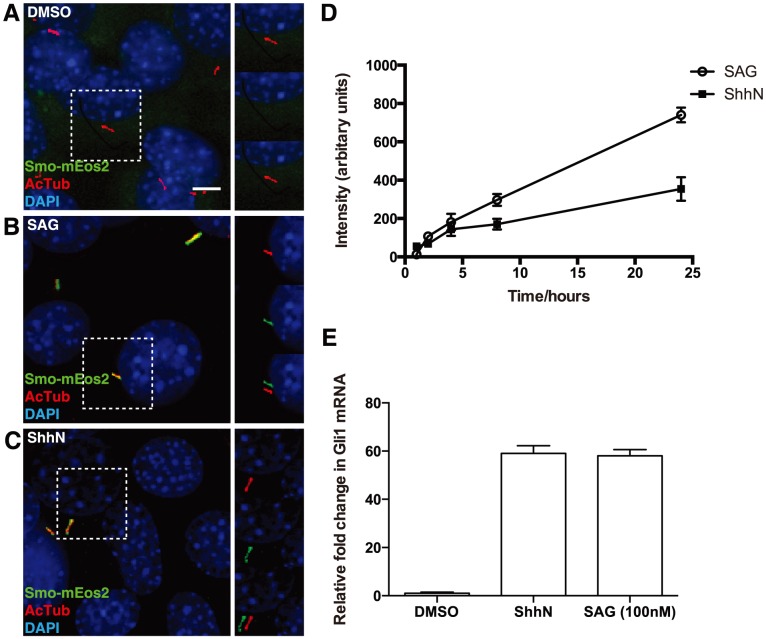
A Hh- and SAG-responsive NIH 3T3/Smo-mEos2 cell line. (A–C) The NIH 3T3/Smo-mEos2 cells were incubated in the absence or presence of SAG or ShhN, and then fixed and stained with anti-acetylated tubulin (primary cilium, red) and DAPI (nucleus, blue). Smo-mEos2 was detected in the primary cilium only when the cells were treated with 100 nM SAG (B) or ShhN (C), but not in the un-treated cells (A). The boxed region in each main panel was viewed on the right in separated red, green channels, and shifted overlays. Scale bar, 5 µm. (D) Mean intensity of Smo-mEos2 fluorescence in cilia of NIH 3T3/Smo-mEos2 cells treated with ShhN or SAG. Each point shows the mean ± SD of fluorescence from 10–20 cilia. (E) The NIH 3T3/Smo-mEos2 cells were incubated in the absence or presence of ShhN or SAG. The mRNA was extracted 48 hours later and real-time quantitative RT-PCR was performed to measure the mRNA level of Gli1.

To simultaneously monitor how the Hh pathway protein Smo enters and exits the primary cilium, we have fused the photoconvertible fluorescent protein mEos2 [16] to the C-terminus of Smo, which permits normal function of the resulting chimeric Smo-mEos2 protein upon introduction into Smo^−/−^ cells (Fig. S1). We generated an NIH 3T3 cell line stably expressing the Smo-mEos2 protein (NIH 3T3/Smo-mEos2), in which the Smo-mEos2 accumulated within cilia in response to Shh or SAG, a small molecule that binds directly to Smo (Fig. 1a–d). The NIH 3T3/Smo-mEos2 cell line shows comparable response to ShhN or SAG as in NIH 3T3 cells (Fig. 1e).

We first treated the NIH 3T3/Smo-mEos2 cells with SAG for 24 hours to allow Smo-mEos2 to accumulate in primary cilia. The cilium highlighted by Smo-mEos2 was selected as a region-of-interest (ROI) for photoconversion with a 405-nm laser. Upon photoconversion, mEos2 shifts from a peak excitation at 506 nm and peak emission at 519 nm (Smo-mEos2 native, hereafter Smo-mEos2^N^), similar to GFP, to a peak excitation at 573 nm and peak emission at 584 nm (Smo-mEos2 photoconverted, hereafter Smo-mEos2^P^), similar to RFP. Both Smo-mEos2^N^ and Smo-mEos2^P^ were subsequently monitored through time-lapse imaging. Thus, an increase of green fluorescence within the cilium reflects Smo-mEos2^N^ entering from the cell body, while a decrease of red fluorescence in the cilium reveals Smo-mEos2^P^ exiting the cilium.

In previous photobleaching experiments, a significant recovery of ciliary fluorescence for the intracellular transport protein IFT88 was detected several minutes after photobleaching [17]. In contrast, we detected little or no recovery of Smo-mEos2^N^ (ciliary entry) 10 minutes after photoconversion (Fig. S2). Similarly, no noticeable reduction of Smo-mEos2^P^ (ciliary exit) was detected within a similar time frame (Fig. S2).

**Figure 2:**
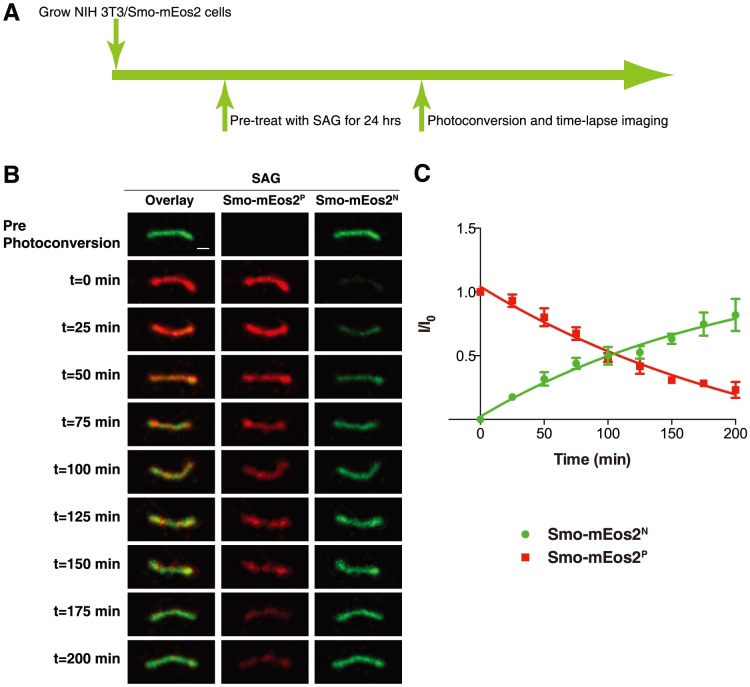
Monitoring ciliary entry and exit of Smo-mEos2 in SAG-treated cells. (A) Schematic diagram of drug treatment and photoconversion of NIH 3T3/Smo-mEos2 cells. (B) Time-lapse images taken at a 25 min interval showing both Smo-mEos2^N^ (green) and Smo-mEos2^P^ (red) from a representative cilium before and after photoconversion at 405 nm. Scale bar, 1 µm. (C) Kinetics of fluorescence recovery of Smo-mEos2^N^ (green) and fluorescence decrease of Smo-mEos2^P^ (red) in the whole cilium of SAG treated cells. Each point shows the mean ± SEM of fluorescence from 5 cilia.

This delay in recovery of ciliary Smo-mEos2^N^ relative to IFT88 may arise from the slower kinetics of Smo entry into the cilium, whether by limited lateral diffusion [18] of membrane proteins from the plasma membrane into the primary cilium, proposed to result from a diffusion barrier at its base [17], or alternatively from the relatively slow process of entry via fusion of internal vesicles [19]. We therefore increased the time-lapse imaging interval and first detected an increased level of Smo-mEos2^N^ in the cilium around 25 minutes after photoconversion. A slight decrease of Smo-mEos2^P^ was also noticed within a similar interval. Using this assay, we observed that Smo-mEos2 proteins constantly enter and exit the cilium in the presence of SAG, with a ciliary retention half-life of approximately 2 hours (Fig. 2).

Given that neither electron microscopic images nor proteome analyses have revealed the presence of proteasomes in cilia [20]–[22], it is unlikely that the observed decrease of Smo-mEos2^P^ is due to its degradation within the primary cilium. We further examined the possible role of protein degradation in turnover of Smo-mEos2 by measuring the stability of Smo-mEos2 protein in cells treated with cycloheximide to block new protein synthesis. We found that Smo-mEos2 in the presence of SAG has a half-life of more than 6 hours (Fig. S3), three-fold longer than the approximate 2-hour ciliary retention half-life of Smo-mEos2 in SAG-treated cells. Therefore, exit of Smo-mEos2 accounts for most, if not all, of its turnover in the primary cilium of SAG-treated cells. Interestingly, we measured a half-life of total Smo-mEos2 protein on the order of 2 hours in cells not treated with SAG (Fig. S3), suggesting that SAG binding to Smo may increase its stability, which is consistent with previous studies showing that SAG-binding to Smo may aid in its maturation [23].

### Ciliobrevin A reduced both ciliary entry and exit of Smo-mEos2

It was previously reported that inactive Smo accumulated in primary cilia of cells with disrupted retrograde intraflagellar transport [11], [15]. We reproduced this ciliary accumulation of Smo in the absence of Hh ligands or Smo agonists in NIH 3T3/Smo-mEos2 cells by adding a low level of ciliobrevin A (CBA) [24], a specific small molecule inhibitor of the minus end-directed microtubule motor cytoplasmic dynein. After 24 hours of treatment with 10 µM CBA, the proportion of ciliated cells was dramatically reduced to about 30% of the entire cell population (Fig. S4C). Smo-mEos2 was detected in more than 70% of these ciliated cells (Fig. S4A, B, D).

**Figure 3:**
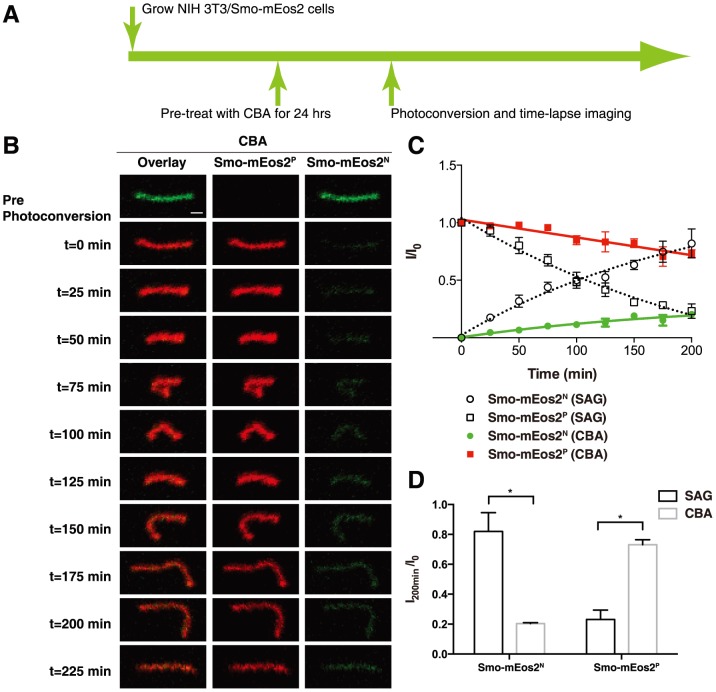
Monitoring ciliary entry and exit of Smo-mEos2 in CBA-treated cells. (A) Schematic diagram of drug treatment and photoconversion of NIH 3T3/Smo-mEos2 cells. (B) Time-lapse images taken at a 25 min interval showing both Smo-mEos2^N^ (green) and Smo-mEos2^P^ (red) from a representative cilium before and after photoconversion at 405 nm. Scale bar, 1 µm. (C) Kinetics of Smo-mEos2^N^ recovery (green) and Smo-mEos2^P^ decrease (red) in the whole cilium from CBA-treated cells. The kinetics of Smo-mEos2^N^ recovery (open circle) and Smo-mEos2^P^ decrease (open square) in the cilium of SAG treated cells were included for comparison. Each point shows the mean ± SEM of fluorescence from 5 cilia. (D) Quantification of Smo-mEos2^N^ recovery (green) and Smo-mEos2^P^ decrease (red) in the cilium of SAG and CBA treated cells. Asterisks show statistically significant differences (Smo-mEos2^N^ recover, P = 0.00059; Smo-mEos2^P^ decrease, P = 0.0035). Error bars show SD, n = 5.

To assay the ciliary entry and exit kinetics of inactive Smo caused by disrupted retrograde intraflagellar transport, we treated the NIH 3T3/Smo-mEos2 cells with 10 µM CBA for 24 hours to allow Smo-mEos2 to accumulate in primary cilia. The cilium highlighted by Smo-mEos2^N^ was selected for photoconversion and both the native and the photoconverted species of Smo-mEos2 were subsequently monitored through time-lapse imaging. When compared with SAG-treated cells, we noticed a much slower entry and exit of both native and photoconverted Smo-mEos2 in the cilium of cells treated with CBA (Fig. 3). Specifically, at 200 minutes after photoconversion, about 80% of Smo-mEos2^P^ remained in the cilium of CBA-treated cells, whereas less than 20% was left in the cilium of SAG-treated cells. Similarly, compared to more than 80% recovery of Smo-mEos2^N^ in the cilium of cells treated with SAG, about 20% of Smo-mEos2^N^ was detected in the cilium of CBA-treated cells.

These results suggest that defects in retrograde intraflagellar transport dramatically slowed down both ciliary entry and exit of Smo. A previous study has demonstrated that ciliobrevins are specific small molecule inhibitors of cytoplasmic dynein, and have no significant effect on kinesin-dependent anterograde microtubule sliding even at a concentration 10 times higher than was used in our experiment [24]. The reduced ciliary exit rate of Smo could be explained as a direct result of the defects in retrograde intraflagellar transport caused by CBA. The reduced ciliary entry rate of Smo in contrast is likely to be an indirect effect of CBA resulting from a cumulative general disruption of ciliary trafficking over time; initial entry is unlikely to be affected, as increase in ciliary Smo was noted when the NIH 3T3/Smo-mEos2 cells were pretreated with SAG for 24 hours, followed by photoconversion and time-lapse imaging immediately after introducing CBA (Fig. S5).

### KAAD-cyclopamine selectively reduces ciliary entry of Smo-mEos2

**Figure 4:**
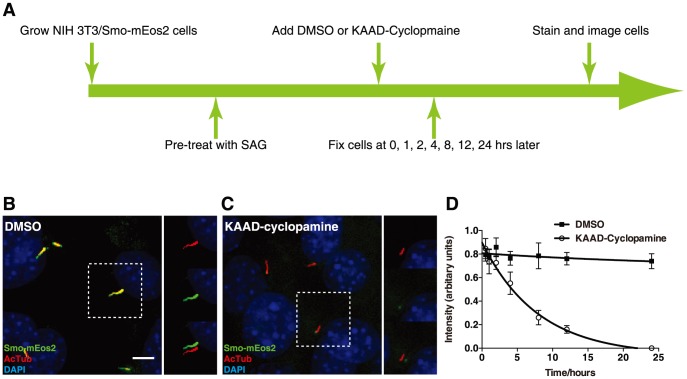
KAAD-cyclopamine blocks SAG induced accumulation of Smo-mEos2 in cilia. (A) Schematic diagram of drug treatment, fixation, and staining of NIH 3T3/Smo-mEos2 cells. (B–C) NIH 3T3/Smo-mEos2 cells were incubated with medium containing 100 nM SAG for 24 h, and then treated with additional 300 nM KAAD-cyclopamine or DMSO (control) for another 24 h followed by fixation, antibody staining, and imaging. Additional KAAD-cyclopamine inhibits accumulation of Smo-mEos2 in cilia of cells induced by SAG (compare B and C). The boxed region in each main panel was viewed on the right in separated red, green channels, and shifted overlays. Scale bar, 5 µm. (D) Mean intensity of Smo-mEos2 fluorescence in cilia of SAG-pre-treated NIH 3T3/Smo-mEos2 cells with addition of DMSO or KAAD-cyclopamine. Each point shows the mean ± SEM of fluorescence from 10–20 cilia.

Ciliary accumulation of Smo has been reported to occur upon treatment with cyclopamine [3]. Curiously, although we and others have confirmed this effect [11], [19], [25], we failed to see Smo accumulation upon treatment with KAAD-cyclopamine (data not shown), a highly potent derivative of cyclopamine [23], [26]. The different effects of cyclopamine and KAAD-cyclopamine on Smo ciliary localization were also found when using our NIH 3T3/Smo-mEos2 cells (Fig. S6). Furthermore, we found in a set of time-series experiments that treatment with 300 nM KAAD-cyclopamine reversed the ciliary accumulation of Smo-mEos2 that is induced by pre-treatment with 100 nM SAG. Specifically, we started to detect reduction of ciliary Smo-mEos2 about 4 hours after adding KAAD-cyclopamine, and ciliary Smo decreased by more than 90% within an additional 8 hours (Fig. 4). To rule out the possibility that the KAAD-cyclopamine-associated removal of ciliary Smo-mEos2 is an artifact due to the mEos2 tag on Smo, we tested the effect of KAAD-cyclopamine on endogenous Smo in NIH 3T3 cells, and found that KAAD-cyclopamine eliminated SAG-induced ciliary accumulation of endogenous Smo (Fig. S7). These observations indicate that the effect of KAAD-cyclopamine on Smo-mEos2 indeed reflects the behavior of wild type Smo proteins.

The observed reversal of ciliary accumulation of Smo-Eos2 could be explained in several ways: (*i*) KAAD-cyclopamine-induced degradation of Smo; (*ii*) decreased ciliary entry rate of Smo; or (*iii*) accelerated ciliary exit of Smo. To distinguish these possibilities, we first used cycloheximide chase experiments to compare the stability of total Smo-mEos2 protein levels in cells treated with SAG vs. SAG followed by addition of KAAD-cyclopamine. Adding KAAD-cyclopamine in cells pre-treated with SAG did not decrease the half-life of Smo-mEos2 (Fig. S8), and instead caused a slight increase in total Smo-mEos2 protein levels as compared to cells treated with SAG alone. We therefore ruled out a destabilizing effect of KAAD-cyclopamine as the cause of Smo-mEos2 ciliary loss.

**Figure 5:**
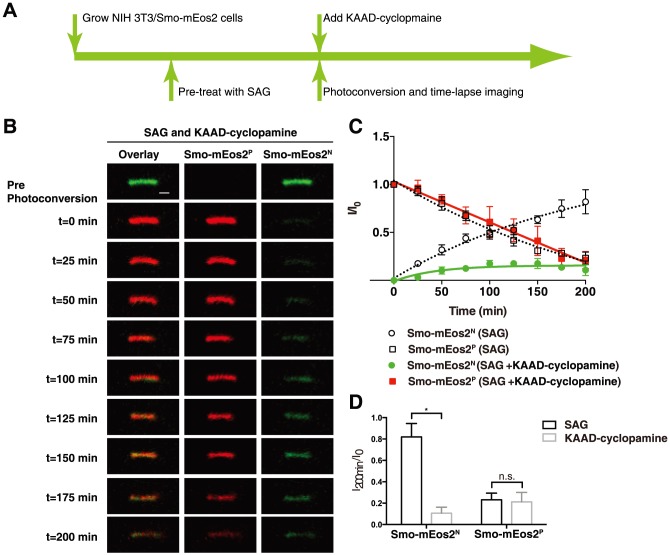
Monitoring ciliary entry and exit of Smo-mEos2 in KAAD-cyclopamine-treated cells. (A) Schematic diagram of drug treatment and photoconversion of NIH 3T3/Smo-mEos2 cells. (B) Time-lapse images taken at a 25 min interval showing both Smo-mEos2^N^ (green) and Smo-mEos2^P^ (red) from a representative cilium before and after photoconversion at 405 nm. Scale bar, 1 µm. (C) Kinetics of Smo-mEos2^N^ recovery (green) and Smo-mEos2^P^ decrease (red) in the whole cilium from KAAD-cyclopamine-treated cells. The kinetics of Smo-mEos2^N^ recovery (open circle) and Smo-mEos2^P^ decrease (open square) in the cilium of SAG treated cells were included for comparison. Each point shows the mean ± SEM of fluorescence from 5 cilia. (D) Quantification of Smo-mEos2^N^ recovery (green) and Smo-mEos2^P^ decrease (red) in the cilium of SAG and KAAD-cyclopamine treated cells. Asterisks show statistically significant differences (Smo-mEos2^N^ recovery, P = 0.0059; Smo-mEos2^P^ decrease, P = 0.86). Error bars show SD, n = 5.

To test for KAAD-cyclopamine triggered changes on ciliary trafficking of Smo-mEos2 we used the photoconversion assay. NIH 3T3/Smo-mEos2 cells were pre-treated with 100 nM SAG for 24 hours to allow Smo-mEos2 to accumulate in primary cilia, and 300 nM KAAD-cyclopamine was added to cells followed by photoconversion of ciliary Smo-mEos2. Both Smo-mEos2^N^ and Smo-mEos2^P^ were subsequently monitored through time-lapse imaging. About 200 minutes after photoconversion, we observed a dramatic reduction of Smo-mEos2^P^ in the cilium of cells exposed to KAAD-cyclopamine, which is similar to the ciliary exit rate of Smo-mEos2 in the cilium of cells treated with SAG alone. In contrast to the normal ciliary exit rate indicated by Smo-mEos2^P^, the ciliary entry rate indicated by recovery of Smo-mEos2^N^ in the cilium of KAAD-cyclopamine treated cells was much slower: thus, at the time when Smo-mEos2^N^ was almost completely recovered in the cilium of SAG treated cells, less than 20% recovery was achieved by Smo-mEos2^N^ in cells treated with SAG and KAAD-cyclopamine (Fig. 5). These data thus suggest that KAAD-cyclopamine-induced loss of Smo from the cilium of SAG treated cells is due to decreased ciliary entry rather than accelerated ciliary exit. Additionally, KAAD-cyclopamine blocked the ciliary accumulation of Smo-mEos2 triggered by CBA (Fig. S9). This result supports the conclusion that KAAD-cyclopamine decreases ciliary entry of Smo-mEos2 even in the absence of agonists. In addition, the finding that blocked ciliary entry of Smo by KAAD-cyclopamine prevents the accumulation triggered by treatment with CBA alone reinforces our conclusion that CBA does not initially block ciliary entry of Smo, and that the entry block we measured after 24 hours of treatment with CBA indeed results from a cumulative overall defect in ciliary trafficking.

Ott and Lippincott-Schwartz [27] recently showed that photoconversion could be used to highlight and track the movement of a subset of molecules within the primary cilium, and Ye et al. tracked the movement of single molecules within the cilium [28]. In both of these studies the major focus was on tracking individual molecules or complexes within the cilium by photoconverting or labeling a small subset of the molecules. Our study differs in that we completely photoconverted a ciliary protein, Smo, in order to measure the ensemble average of ciliary entry and exit kinetics. This required relatively long intervals for time-lapse imaging as well as a long imaging duration in order to capture the slow overall kinetics of ciliary entry and exit of transmembrane proteins such as Smo.

In summary, we have established a live-cell imaging assay that allows a separate but simultaneous examination of ciliary entry and exit of Smo in individual cells. Using this assay, we find that activated Smo constantly enters and exits the cilium with a ciliary retention half-life of approximately two hours. We also find that small molecules such as KAAD-cyclopamine selectively block ciliary entry of Smo, whereas CBA interferes initially with ciliary exit but eventually with both ciliary entry and exit of Smo. Our study provides an approach to understanding regulation of ciliary entry vs. exit of Hedgehog pathway components, as well as other ciliary proteins.

## Methods

### Cell culture, Constructs and Reagents

mEos2 (Addgene plasmid 20341) was fused to mouse Smo at the C terminus to generate the Smo-mEos2 construct. The stable cell line expressing Smo-mEos2 was produced by site-specific recombination into a single site in the genome of 3T3 cells using the Flp-In system (Invitrogen). We obtained SAG from Enzo Life Sciences, KAAD-cyclopamine from EMD Chemicals, CBA, Cycloheximide and DMSO were from Sigma. ShhN conditioned medium were prepared as previously described [29].

### Antibodies

Antibodies were used at the following concentrations: rabbit anti-Smo antibody [11] 1∶1000, mouse monoclonal anti-acetylated tubulin antibody (Sigma) 1∶2000, mouse anti-beta tubulin (Developmental Studies Hybridoma Bank) 1∶5000, secondary antibodies (Jackson ImmunoResearch Laboratories) 1∶500.

### RT-PCR

NIH 3T3/Smo-mEos2 cells were grown to reach confluence. The cells were then shifted to 0.5% serum medium and incubated 24 h with or without ShhN or SAG as described. RNA from the cells was extracted and purified using the RNeasy Mini Kit from Qiagen. One-step RT-PCR was performed using the OneStep RT-PCR kit (Qiagen) on the 7300 Real-Time PCR System (Applied Biosystems). Non-linear regression analysis for curve fitting was performed using GraphPad Prism software.

PCR primers were as follows: 5′- AAGGAATTCGTGTGCCATTGGG-3′ and 5′- ACATGTAAGGCTTCTCACCCGT-3′ for Gli1, 5′- CGTGATTAGCGATGATGAACCAGG-3′ and 5′- CATCTCGAGCAAGTCTTTCAGTCC-3′ for HPRT1 (internal reference).

### Cycloheximide chase and Western blot analysis

NIH 3T3/Smo-mEos2 cells were grown to confluence and shifted to 0.5% serum medium and treated with DMSO or SAG for 24 h before the addition of cycloheximide (in the continued presence of DMSO, SAG, or KAAD-cyclopamine as described in related experiments) to block protein synthesis for the indicated periods of time. Cells were lysed and the level of total Smo-mEos2 was measured by immunoblotting.

### Gli reporter assays

NIH 3T3/Smo-mEos2 cells were plated at 5–9×10^4^ cells/well of 24-well plates and transfected the next day using FuGENE HD (Promega) with Gli-luciferase reporter, control pRL-SV40 renilla luciferase, and other DNA constructs as indicated. After cells reached confluence in about 2 days, they were shifted to 0.5% serum medium and incubated 24 h with ShhN.

### Immunofluorescence and Quantification of Microscopic images

Cells were fixed in 4% formaldehyde for 10 min, and then washed 3 times with PBS. Fixed cells were placed in blocking solution (PBS with 1% normal goat serum and 0.1% Triton X-100) for 30 min. Primary antibodies were diluted in blocking solution and used to stain cells for 1 h at room temperature. After 3 washes in PBS, secondary antibodies and DAPI (Invitrogen) were added in blocking solution at a dilution of 1∶500 for 1 h at room temperature. The samples were mounted in VECTASHIELD Mounting Medium (Vector Laboratories) for microscopy. Microscopy was performed on a Leica spinning disc confocal microscope SD6000. Images were taken with a 63× objective. All analysis was performed using ImageJ, as described previously [3]. For the quantitative analysis of Smo-mEos2 levels in primary cilia, all images used for comparisons within an experiment were obtained with identical settings on the microscope and then used for quantification without any manipulation. A mask was constructed by manually outlining cilia in the image taken in the acetylated-tubulin channel. This mask was then applied to the image taken in the Smo-mEos2 channel, and the fluorescence at the cilia was measured. Local background correction was performed by moving the mask to measure fluorescence at a representative nearby region, and then subtracting this value from that of ciliary fluorescence. All points represent mean (±SEM) fluorescence from 10–20 individual cilia. Statistical analysis was performed using GraphPad Prism software.

### Live cell imaging and Photoconversion

NIH 3T3/Smo-mEos2 cells cultured in glass bottom dishes (In Vitro Scientific, Sunnyvale, CA, USA) were grown to reach confluence. The cells were then shifted to 0.5% serum medium and incubated 24 h with SAG or other chemicals as described before imaging. As described previously (Yu et al., 2011), imaging of live cells was performed with a Zeiss LSM510 Meta inverted confocal microscope equipped with the Zen 2009 software and an environmental control chamber (5% CO_2_, 37°C and humidity). A cilium was selected and focused on in the green channel (Smo-mEos2^N^), which was outlined as the region of interest for photoconversion. Smo-mEos2 conversion was achieved by the scanning of outlined primary cilium with a 405 nm laser (2% intensity) for 10 iterations using a 63× objective and 4× Zoom. Time-lapse recordings of both Smo-mEos2^N^ channel (excitation with 488/emission BP505–550) and Smo-mEos2^P^ channel (excitation with 561/emission LP575) were performed every 1 or 25 min as described. Images were exported from Zen 2009 and analyzed with ImageJ. For the quantitative analysis of Smo-mEos2 levels in primary cilia, all images used for comparisons within an experiment were obtained with identical settings on the microscope and then used for quantitation without any manipulation. A mask was constructed by manually outlining cilia in the image taken in the Smo-mEos2^N^ channel before photoconversion and in the Smo-mEos2^P^ channel after photoconversion. This mask was then applied to the image taken in the Smo-mEos2^P^ channel before photoconversion and in the Smo-mEos2^N^ channel after photoconversion, respectively. The fluorescence at the cilia at different time points was measured. Local background correction was performed by moving the mask to measure fluorescence at a representative nearby region, and then subtracting this value from that of ciliary fluorescence. All points represent mean (SEM) fluorescence from 4–5 individual cilia. Statistical analysis was performed using GraphPad Prism software.

## Supporting Information

Figure S1 **Smo-mEos2 shows normal function upon introduction into Smo^−/−^ cells.** Smo^−/−^ cells were transiently transfected with Smo-mEos2, Gli-luciferase reporter, and control SV40-Renilla luciferase. Following transfection, cells were grown to confluency, incubated with ShhN, and assayed for reporter activity. Error bars indicate SD, n = 3.
(EPS)

Figure S2 **Monitoring ciliary entry and exit of Smo-mEos2 in SAG-treated cells.** (A) Schematic diagram of drug treatment and photoconversion of NIH 3T3/Smo-mEos2 cells. (B) Time-lapse images taken at 75-second intervals showing both Smo-mEos2^N^ (green) and Smo-mEos2^P^ (red) from a representative cilium before and after photoconversion at 405 nm. Scale bar, 1 µm.
(EPS)

Figure S3 **Cycloheximide chase experiments to measure the half-life of total Smo-mEos2 protein in cells treated with SAG.** (A) NIH 3T3/Smo-mEos2 cells were induced with (right panel) or without (left panel) SAG for 24 h before the addition of cycloheximide (right panel, in the continued presence of SAG) to block protein synthesis for the indicated periods of time. The level of total Smo-mEos2 was measured by immunoblotting. (B) Quantification of normalized (by beta-tubulin) Smo-mEos2 protein levels from cells treated with cycloheximide for the indicated periods of time.
(EPS)

Figure S4 **Smo accumulated in primary cilia of cells treated with CBA.** (A–B) NIH 3T3/Smo-mEos2 cells were incubated in the absence or presence of CBA for 24 h, and then fixed and stained with anti-acetylated tubulin (primary cilium, red) and DAPI (nucleus, blue). Smo-mEos2 was detected in the primary cilium only when the cells were treated with 10 µM CBA (B), but not in the un-treated cells (A). The boxed region in each main panel was viewed on the right in separated red, green channels, and shifted overlays. Scale bar, 5 µm. (C) Quantification of the percentage of ciliated cells 24 h after treatment with either CBA or SAG. (D) Quantification of the percentage of cilium with Smo-mEos2 24 h after treatment with either CBA or SAG.
(EPS)

Figure S5 **Monitoring ciliary entry and exit of Smo-mEos2 in SAG, followed by CBA-treated cells.** (A) Schematic diagram of drug treatment and photoconversion of NIH 3T3/Smo-mEos2 cells. (B) Time-lapse images taken at a 25 min interval showing both Smo-mEos2^N^ (green) and Smo-mEos2^P^ (red) from a representative cilium before and after photoconversion at 405 nm. Scale bar, 1 um.
(EPS)

Figure S6 **Ciliary accumulation of Smo-mEos2 upon treatment with inhibitory concentrations of cyclopamine, but not KAAD-cyclopamine.** NIH 3T3/Smo-mEos2 cells were incubated with medium containing 3 µM of cyclopamine (A) or 300 nM of KAAD-cyclopamine (B) for 24 h followed by fixation, antibody staining, and imaging. The boxed region in each main panel was viewed on the right in separated red, green channels, and shifted overlays. Scale bar, 5 µm.
(EPS)

Figure S7 **KAAD-cyclopamine blocks SAG induced accumulation of endogenous Smo in cilia.** (Top) Schematic diagram of drug treatment, fixation, and staining of NIH 3T3 cells. (A–C) NIH 3T3 cells were incubated with medium containing DMSO, SAG, or SAG followed by 300 nM of KAAD-cyclopamine before fixation, antibody staining, and imaging. Addition of KAAD-cyclopamine inhibits accumulation of Smo in cilia of cells induced by SAG (compare C to B). The boxed region in each main panel was viewed on the right in separated red, green channels, and shifted overlays. Scale bar, 5 µm.
(EPS)

Figure S8 **Cycloheximide chase experiments to measure the half-life of Smo-mEos2 in cells treated with SAG followed by additional KAAD-cyclopamine.** (A) Schematic diagram of drug treatment and immunoblotting of NIH 3T3/Smo-mEos2 cells. (B) NIH 3T3/Smo-mEos2 cells were induced with SAG for 24 h before the addition of DMSO (upper panel) or KAAD-cyclopamine (lower panel) together with cycloheximide in the continued presence of SAG to block protein synthesis for the indicated periods of time. The level of total Smo-mEos2 was measured by immunoblotting. (C) Quantification of normalized (by beta-tubulin) Smo-mEos2 protein levels from cells treated by DMSO or KAAD-cyclopamine with cycloheximide for the indicated periods of time.
(EPS)

Figure S9 **KAAD-cyclopamine blocks the ciliary accumulation of Smo-mEos2 triggered by CBA.** (Top) Schematic diagram of drug treatment, fixation, and staining of NIH 3T3/Smo-mEos2 cells. (A–D) NIH 3T3/Smo-mEos2 cells were incubated with medium containing DMSO (control), CBA, KAAD-cyclopamine, or a combination of CBA and KAAD-cyclopamine for 24 h followed by fixation, antibody staining, and imaging. The boxed region in each main panel was viewed on the right in separated red, green channels, and shifted overlays. Scale bar, 5 µm.
(EPS)
